# Benthic estuarine communities' contribution to bioturbation under the experimental effect of marine heatwaves

**DOI:** 10.1038/s41598-021-90720-7

**Published:** 2021-06-01

**Authors:** M. Dolbeth, O. Babe, D. A. Costa, A. P. Mucha, P. G. Cardoso, F. Arenas

**Affiliations:** 1 CIIMAR - Interdisciplinary Centre of Marine and Environmental Research, Novo Edifício Do Terminal de Cruzeiros Do Porto de Leixões, Avenida General Norton de Matos S/N, 4450-208 Matosinhos, Portugal; 2 Center of Exact and Nature Sciences (CCEN), Department of Systematics and Ecology (DSE), UFPB - Federal University of Paraíba, Jardim Cidade Universitária s/n, João Pessoa, 58051-090 Brazil

**Keywords:** Biodiversity, Climate-change ecology, Community ecology

## Abstract

Marine heatwaves are increasing worldwide, with several negative impacts on biological communities and ecosystems. This 24-day study tested heatwaves' effect with distinct duration and recovery periods on benthic estuarine communities' diversity and contribution to ecosystem functioning experimentally. The communities were obtained from a temperate estuary, usually subjected to high daily thermal amplitudes. Our goal was to understand the communities' response to the thermal change, including the community descriptors and behavioural changes expected during heat extremes. We measured community composition and structural changes and the bioturbation process and nutrient release as ecosystem functioning measurements. Overall, our findings highlight the potential tolerance of studied estuarine species to the temperature ranges tested in the study, as community composition and structure were similar, independently of the warming effect. We detected a slight trend for bioturbation and nutrient release increase in the communities under warming, yet these responses were not consistent with the heatwaves exposure duration. Overall, we conclude on the complexity of estuarine communities’ contribution to functioning under warming, and the importance of scalable experiments with benthic organisms' responses to climate variability, accommodating longer time scales and replication. Such an approach would set more efficient expectations towards climate change mitigation or adaptation in temperate estuarine ecosystems.

## Introduction

All over the world, marine heatwaves are increasing in intensity, duration, and frequency at a dramatic rate^1–3^. Due to their nature, heatwaves are extreme climate events, as they represent sudden and abnormal temperature increases that may last from 5 consecutive days to even months^4^. Marine ecosystems are particularly vulnerable to heatwaves, with documented impacts that range from species physiology aspects^5–7^ to broader consequences at communities^8–10^ and ecosystem-level^2,9,11^, with severe economic implications^12,13^.

Among known impacts of marine heatwaves are the physiological adjustments to protect and repair the individual internal structure due to thermal stress^6,7^ or behavioural ones (e.g. bioturbation to avoid the heat^14^). At the population level, changes in growth, reproduction^15,16^ or survival^14,17^ may be expected. For communities, displacement might occur to avoid adverse temperature and mass mortality^10,18^. Also, impacts of marine heatwaves combined with other anthropogenic impacts place several regions of the world at risk of species distribution shifts and decline in biodiversity and its sustained ecosystem services^3^. Yet, the sort and scale of such impacts depend on the duration, magnitude and timing of the heatwave^19^, and nature of the ecosystem itself and affected biological communities, i.e., their sensitivity and adaptive capacity^3,11^. For instance, estuarine fauna is generally well adapted to changes in temperature^20^, including a wide daily temperature amplitude, because several species are intertidal and partially exposed to air during the tidal cycle^21^. One could speculate on their ability to cope with extreme temperature events^14,22^. However, negative impacts are likely to occur when species face prolonged periods of temperature increase that exceed their tolerance levels, without a possibility of recovery (i.e., back to tolerable temperature levels^7^). Empirical data from field studies on the impacts of heatwaves in estuarine ecosystems confirms several negative consequences, threatening diversity^23^, affecting ecosystem functioning (e.g., biological production decline^24^), and ultimately the ecosystem services underpinning human wellbeing^3^.

Despite the evidence from empirical data, species-environment interactions and their implications for the marine ecosystem functioning are unpredictable regarding temperature extremes effects. Behavioural changes are expected^25,26^, which for benthic fauna, changes in bioturbation may be of particular concern, influencing biogeochemical cycles, oxygenation^27–29^ and preventing sediment erosion^30^ on the one hand, while contributing to destabilising the sediment structure if unbalanced, on the other hand^27,30–32^. Manipulative experiments provide a valuable tool because they allow exploring how species-environment interactions change under environmental stress and directly measure the change in ecosystem processes and functions^33,34^. Such an approach would contribute to a broader understanding of the implications of climate change in marine communities.

Most experimental studies testing temperature impact have focused on particular species or communities with different species compositions to define species thermal tolerances and limits^14^ or unravel potential effects on specific aspects of ecosystem functioning^26,34,35^. Existing experimental works studying the effects of temperature extremes oscillations (by simulating heatwaves) have detected different, often contrasting, biological responses, ranging from a lack of effect, negative or positive effect depending on species tolerance, species interactions, and local context^19,36,37^. Also, a particular species or community's biological responses may change depending on whether a single or multiple and sequential heatwaves occur^37^. We still lack a better understanding of what happens after the heat occurrence (i.e., recovery period) and what may be driving species resistance or resilience to the warm from an ecological perspective. These studies acquire particular relevance as heatwaves frequency, intensity, and duration will increase in the future^1,3^.

This study aims to understand the impact of heatwaves, with different duration and recovery periods, on the biodiversity patterns and ecosystem functioning of estuarine communities, particularly on the bioturbation process and nutrient release. We used macrofauna benthic communities, which are known to mediate bioturbation and affect/promote other functions related to biogeochemical cycles^28,38,39^. We also focus on estuarine communities from a temperate region, usually subjected to high daily thermal amplitudes, to understand their response to thermal change. We want to know the effect of the heatwave on benthic communities and whether the heatwave duration and temperature drop to previous levels can influence the community responses in general.

## Results

### Bioturbation and nutrient dynamics

**Figure 1:**
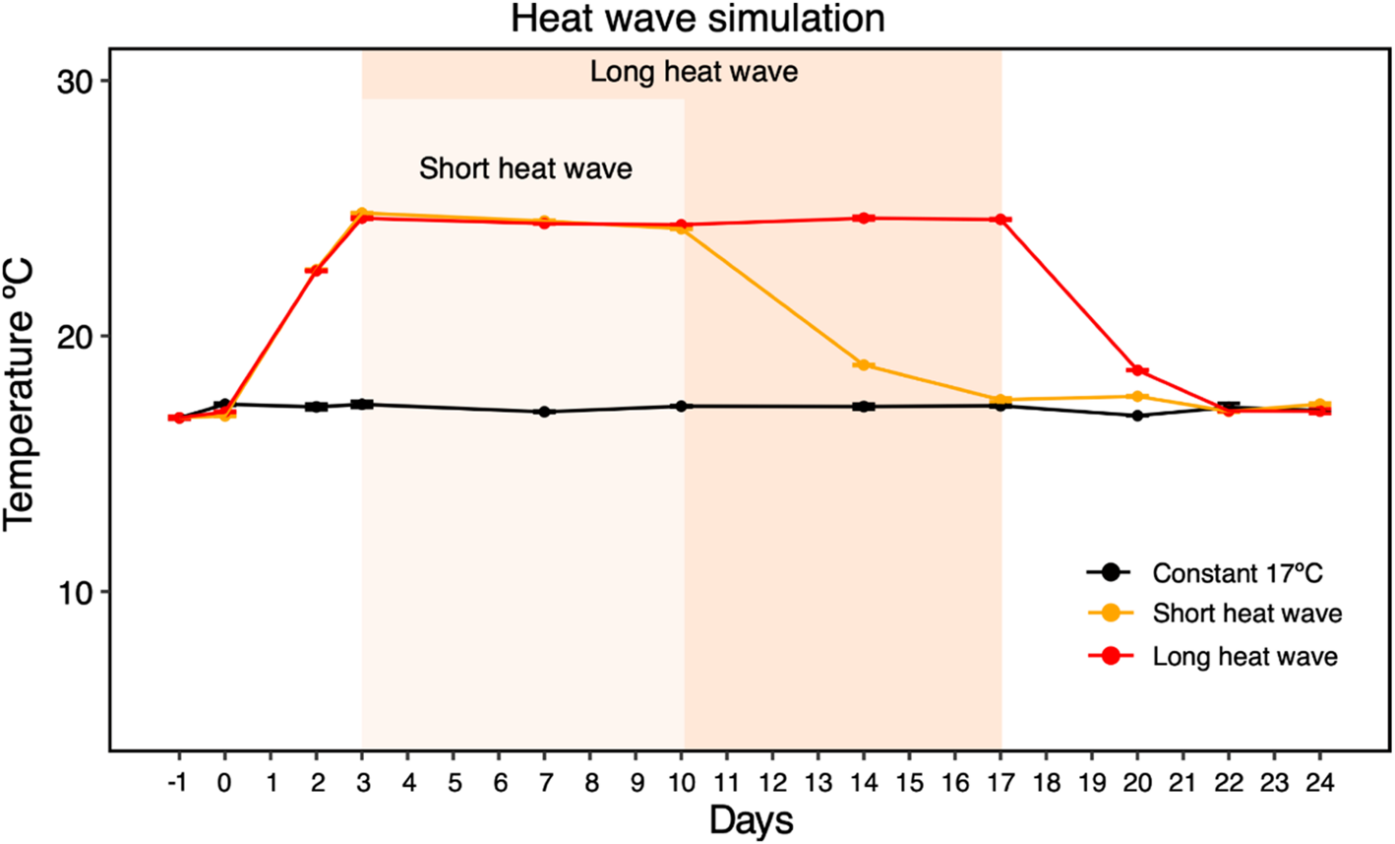
Details of the three temperature experimental conditions, with the measured temperature (mean and standard deviation) during the experimental procedure, highlighting the heatwave simulation periods for each temperature treatment. Graph done with ggplot2 from R software (https://ggplot2.tidyverse.org) and shadow included with INKSCAPE (https://inkscape.org/).

All temperature treatments were reproduced as planned in the experimental design (Fig. 1). Oxygen salinity and pH varied within similar values along with the whole experimental procedure (77.4 ± 4.29%, 34.1 ± 0.83 and 8.1 ± 0.15, respectively).

**Figure 2:**
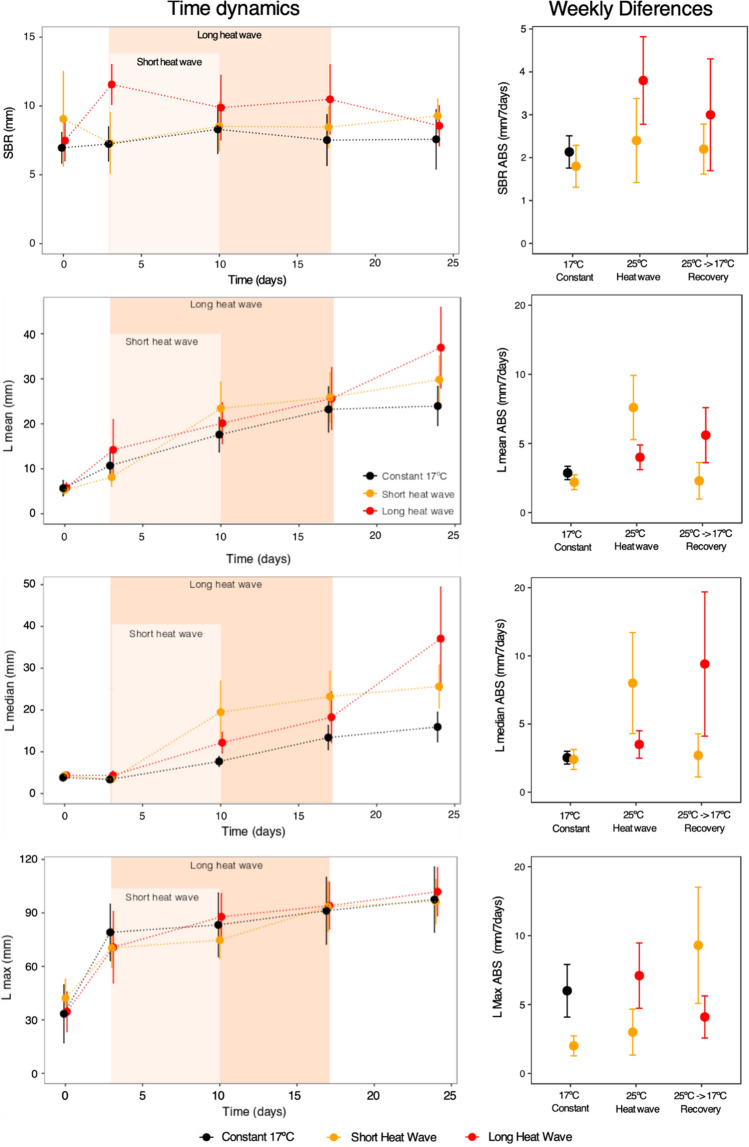
Particles reworking variables' variation throughout the experimental procedure, considering the three temperature treatments. Left side, temporal dynamics with the indication of the short heatwave and long heatwave periods; right side, the absolute difference for every seven days, considering the constant (17 °C), heatwaves (25 °C) and recovery period, when temperature declined from 25 °C to 17 °C. Graphs done with ggplot2 from R software (https://ggplot2.tidyverse.org) and figure compiled with INKSCAPE (https://inkscape.org/).

All particle reworking variables measured as a representation of the bioturbation process were time-dependent (p-perm = 0.001, Table 2S), except for SBR. No significant differences were found between temperature treatments (Fig. 2, Table 2S). A bioturbation increase over time was linear/constant for Lmean and Lmedian, with differences practically between all sampling times (p-perm < 0.05), except between days 17 and 24 (p-perm > 0.05, Table 2S). For Lmax, which is the maximum particle reworking of the organisms in a community, particle reworking increased during the first three days of the experiment (p-perm < 0.02, Table 2S) but not on the subsequent sampling dates (p-perm > 0.05, Table 2S, Fig. 2). Despite no significant differences found for the temperature treatments (p-perm > 0.05, Table 2S), Lmedian was slightly lower in the constant temperature treatment compared to the others and was marginally higher for the short heatwave except for the last day (Fig. 2). The variation trend was similar for Lmean, yet less pronounced, while for Lmax and SBR, differences among temperature treatments were even less evident.

When analysing the particle reworking data as the absolute weekly difference, behavioural trend differences between temperature treatments became more evident, particularly for Lmean and Lmedian (Fig. 2). For the weeks when the temperature was 17 °C (only available for constant and short heatwave treatments), particle reworking varied within similar values in general (Fig. 2). During the heatwave simulation, when the temperature remains at 25 °C, Lmean and Lmedian generally were higher for the short heatwave treatment than the 17 °C temperature week and the recovery week, when the temperature dropped from 25 °C to 17 °C (Fig. 1). The opposite was observed for the long heatwave (Fig. 2). During the recovery week, Lmedian and Lmean drop to values similar to those observed at 17 °C for the short heatwave but were higher for the long heatwave treatment. These results were only significant for Lmedian (p-perm = 0.015, Table 2S) and Lmean (p-perm = 0.01, Table 2S).

**Figure 3:**
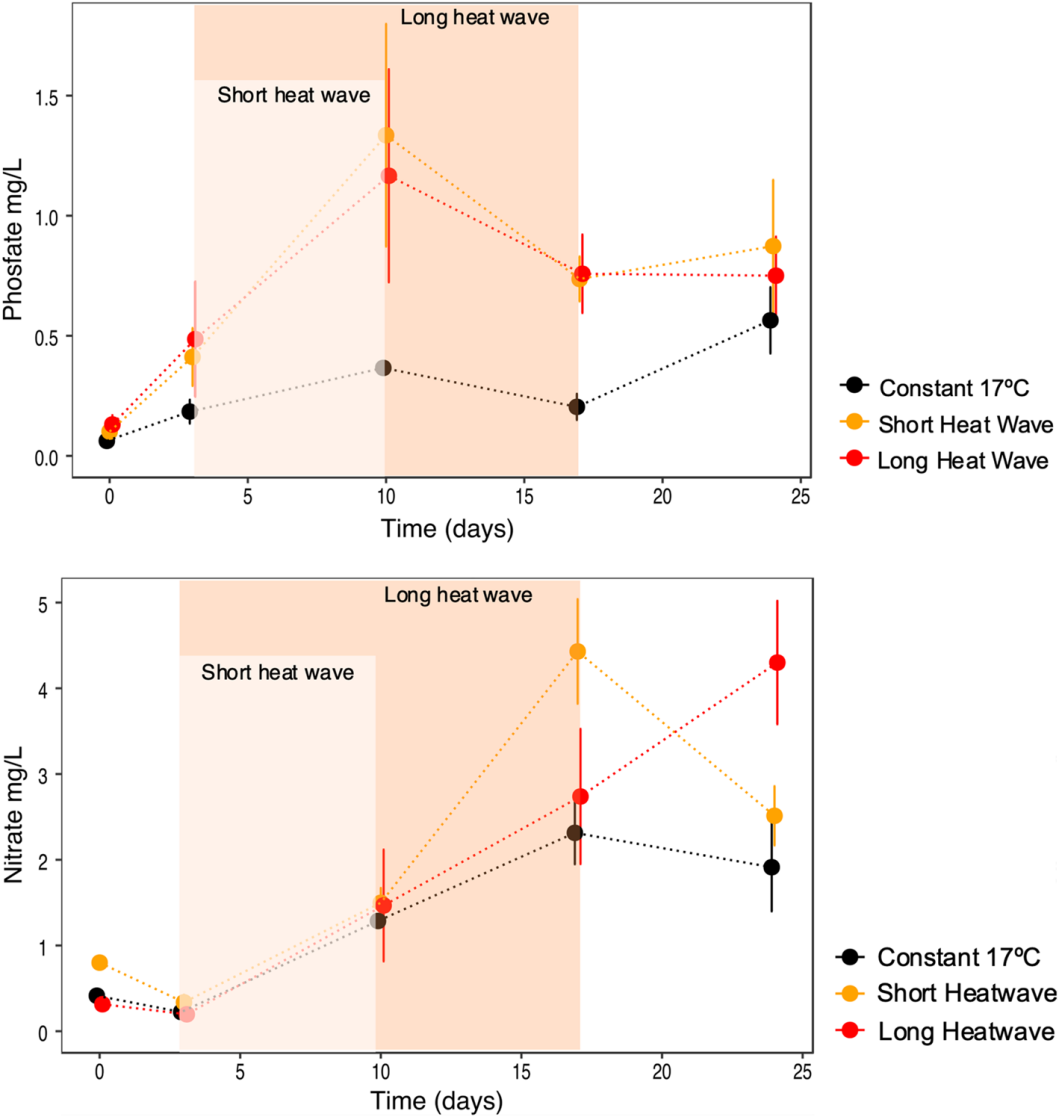
Phosphate and nitrate concentrations in the water column throughout the experimental procedure, considering the three temperature treatments. Graphs done with ggplot2 from R software (https://ggplot2.tidyverse.org) and figure compiled with INKSCAPE (https://inkscape.org/).

Regarding nutrients, different patterns emerged depending on the nutrient identity. Phosphates release was higher for the samples subjected to a temperature increase, independently of their duration (significant differences between control vs the temperatures treatments, p-perm < 0.03 Table 2S, Fig. 3). For nitrates, differences between treatments were only visible after the heatwave occurrence (significant interaction between treatment and time: p-perm = 0.045, Table 2S, Fig. 3), i.e., in the recovery week when temperature drops from 25 to 17 °C (Fig. 1). During this week, nitrates release increased for both temperature treatments (at day 17 for the short heatwave and day 24 for the long heatwave treatment, Fig. 3). Values then drop when temperature stabilises at 17 °C to values similar to those of the constant treatment, but this was only possible to observe for the short heatwave treatment (Fig. 3). These pairwise differences were not confirmed statistically due to the low number of replicates (and permutations) tested in the significant interaction (Table 2S).

### Benthic communities

**Figure 4:**
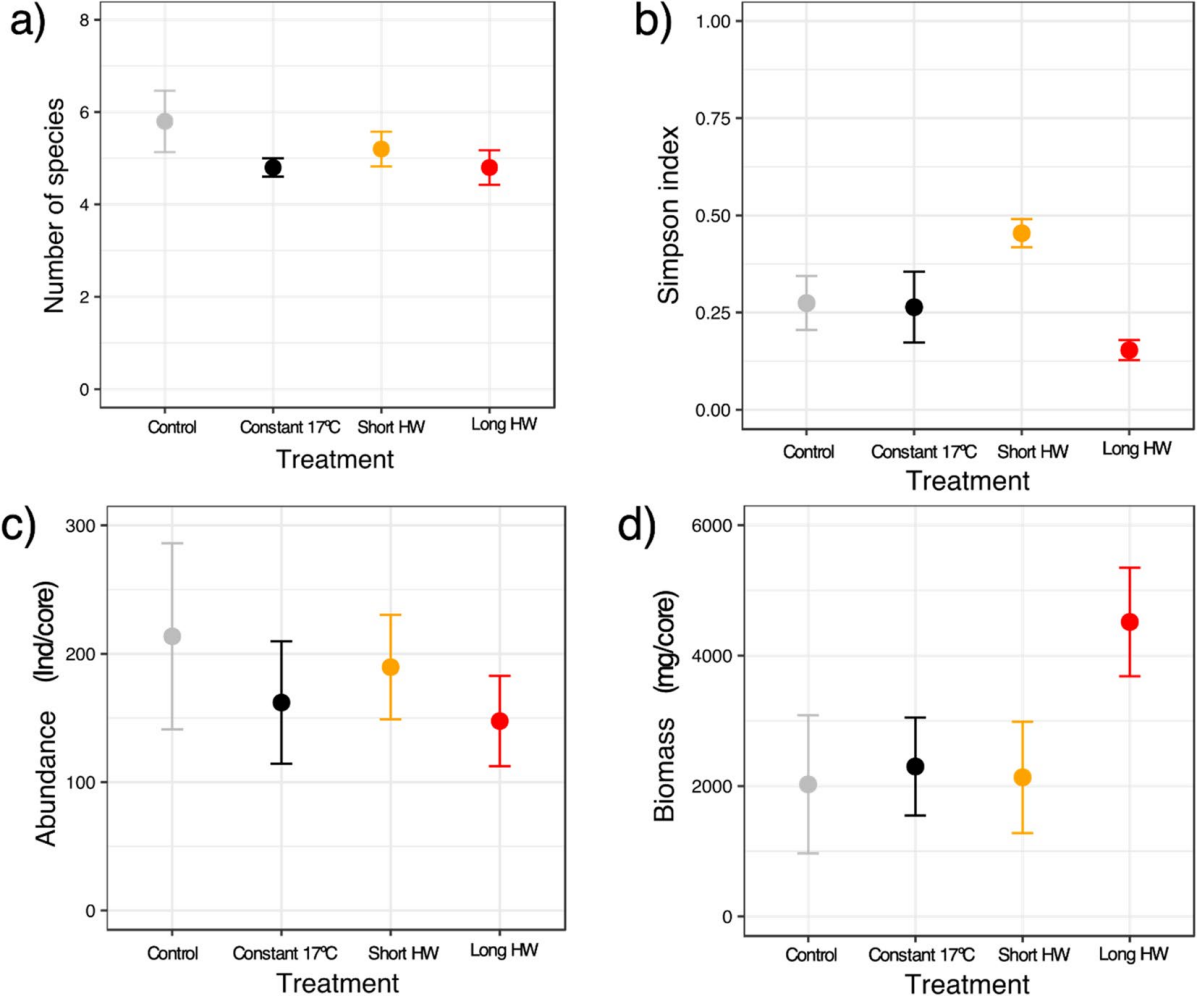
Macrobenthic community data (mean and standard deviation) for the initial control samples and each temperature treatment (symbols' colours), considering (**a**) species number, (**b**) Simpson index measured with biomass, (**c**) community abundance (number of individuals per core), (**d**) community biomass (mg ash-free dry mass—AFDM per core). Graphs done with ggplot2 from R software (https://ggplot2.tidyverse.org) and figure compiled with INKSCAPE (https://inkscape.org/).

Analyses on the benthic communities before and after the experimental procedure confirmed no differences when comparing species number, varying between 4 and 8 species, and total community abundance and biomass per aquaria between treatments (Fig. 4, ANOVA p > 0.05, Table 3S). Despite the highest community biomass mean values apparent for the long heatwave treatment compared to the other treatments (Fig. 4), results were still not significantly different (ANOVA p > 0.05, Table 3S). However, when analysing the Simpson index measured with species biomass, differences were significant, with the highest value for the short heatwave and lowest for the long one treatment (ANOVA p = 0.015, Fig. 4).

**Figure 5:**
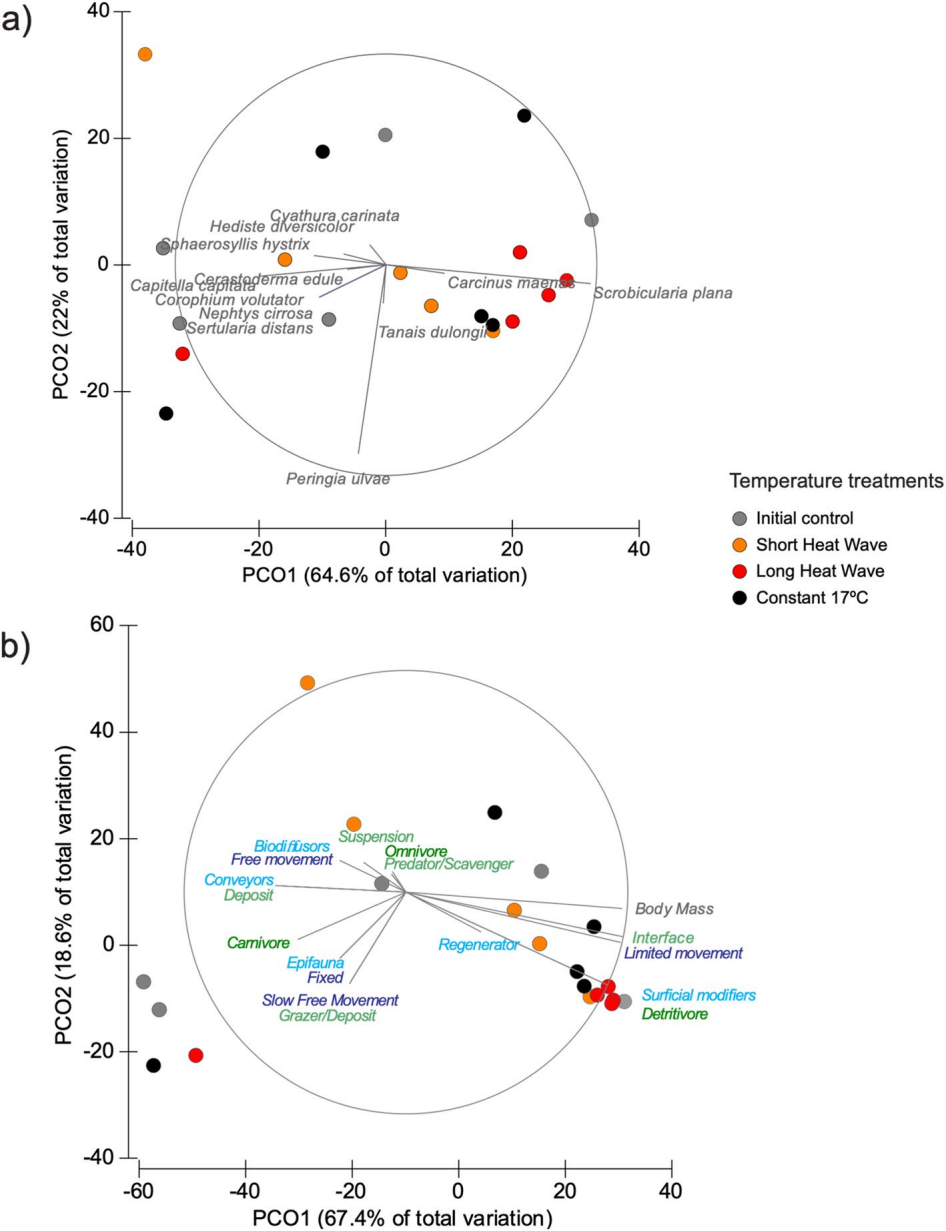
PCO ordination for (**a**) macrobenthic community biomass and (**b**) community traits weighted by biomass for each temperature treatment (symbols' colours). Vectors based on the spearman correlations between species or trait category and PCO axes were superimposed on the plot for guidance in the interpretation. Graphs done with PRIMER v6 with PERMANOVA add-on and figure compiled with INKSCAPE (https://inkscape.org/).

Again, we did not found differences for species biomass composition and structure between treatments (pperm = 0.719, Table 3S), despite the trend for long heatwave treatment samples to cluster (Fig. 5a). The species *Scrobicularia plana* was associated with 4 of those aquaria (Fig. 5a), with this species having a larger body mass in general (Table 1S). The composition differences were also expressed in the functional organisation of communities, taking into account the five traits, since four of the long heatwaves aquaria also clustered, despite not statistically significant (pperm = 0.532, Table 3S). These samples were associated with larger individuals, mostly surficial modifiers, with limited movement, that could be interface feeders (Fig. 5b).

Overall, surficial modifiers (from the sediment reworking trait) and detritivore species (from the trophic group trait) were dominant in all communities, accounting for more than 93% of the community biomass (community weighted mean analyses, Table 4S). Yet, for the other traits, most organisms varied within the limited movement and slow, free movement for the trait mobility, and their feeding changed within grazer/deposit or interface feeders (i.e., switching between suspension and deposit-feeding depending on local conditions). Regarding the average individual body mass, values were generally higher for the long heatwave treatment, except in one of the replicates (Table 4S).

## Discussion

Temperature is a trigger of several essential ecological processes, yet it can also have dramatic consequences when exceeding species thermal range, such as during a heatwave occurrence^17,24^. In this study, we tested the effect of heatwave occurrence, duration and recovery on estuarine benthic communities’ diversity and behaviour. Since diversity decline and changes in estuarine ecosystem functioning have been documented during marine heatwaves^23,39^, and we used entire communities directly from the field, we expected to quantify diversity and other functional changes. However, our results were inconclusive regarding the warming effect, despite a slight trend for increasing bioturbation and nutrient release compared to a control. We also did not find significant changes in benthic communities' general descriptors (e.g., diversity, community structural and functional composition). Next, we expand on these main findings.

All particle reworking variables characterising bioturbation, apart from the surface boundary roughness, showed an increasing trend during the experimental period. This increase was expected since the muddy sediments favour bioturbation and sediment resuspension^29,30^, and due to the arrival to a new environment, consequent tube-building, sediment prospecting^25^ and its cumulative effect. Lmax dynamics in the first days of the study evidenced the arrival to the aquaria (i.e., artificial starting point due to the beginning of the quantification), which then stabilised to a maximum depth. However, there were no evident differences among temperature treatments, despite the slight trend for lower values in the constant temperature treatment. Other works from this geographical region, with mild (up to 2 °C) and surficial temperature increases with estuarine communities in situ*,* were also unable to conclude the impact of temperature on bioturbation^40^. Since we tested an 8 °C sudden increase, we would expect more expressive differences in the community behaviour, which could encompass activity decrease as a response to temperature increase stress within a non-lethal threshold^14,41,42^ or a vertical movement towards deeper sediment areas^16^. Also, recent studies relate the intensity of bioturbation to the species metabolism, which is temperature-sensitive^39,43,44^, reinforcing our initial hypothesis to expect differences due to the warming. However, the community response would be influenced by density-dependent factors and potential species interactions^45,46^, which may hinder the detection of a clear pattern.

Our results may express the tolerance of these estuarine species to the temperature oscillations represented in the study and the competitive balance in the species' behaviour that compose the communities^37,47^, which may confer some resistance to the heat effect. Indeed, these species have a wide distribution and are common in intertidal mudflats, being exposed to air temperature. For this southern temperate region, some of its dominant species may thrive in temperature ranges within 15 °C to 23 °C, tolerating up to 28 °C^14^, which is within the tested thermal range. Also, some might activate internal protective mechanisms that allow coping with the short-term warming, as concluded for *Scrobicularia plana*, under the same environmental conditions of this study^22^. When analysing bioturbation data as weekly differences, which would showcase any different behaviour regarding the heat impact, a different pattern emerged, but also not conclusive as the variation patterns were opposite for the heatwave treatments. While particle reworking increased during the short heatwave, it maintained similar values of the constant temperature (control) for the long heatwave. Once the temperature dropped from 25 °C to 17 °C, sediment reworking increased only for the long heatwave. These no consistent temperature effects could be random or result from species composition and functional differences (discussed below).

Bioturbation mediated by macrofauna generally increases sediments' oxygenation and may be an essential trigger for nutrient release^35,39,48,49^. However, the variation trends in the nutrient release data in this study were not directly correlated with macrofauna's bioturbation, as also concluded by^33,40^. Sediment–water nutrient exchanges reflect complex interactions with several factors, as microbiota, sediment composition, physico-chemical environment, and benthic composition, including bioturbation^33,34,48^. Both nutrients concentrations increased over time, to values still within^50^ or below^51^ the range of other experiments. Phosphate release increased during the temperature increase, as observed before in field conditions for our estuarine sediments and biological donor communities^52,53^. However, we could not detect a different result due to the heatwave duration, as values were similar for both heatwave treatments. Regarding nitrates, results suggest a delay in response to the warming, with an increasing nitrate release 1-week after the heatwave occurrence. However, we could not relate it with the bioturbation, nor with the specific functional behaviour of the species that compose the benthic community, as in^35^. We would need to extend the experiment to conclude some warming effect, as the impact of warming in species' bioturbation and nutrient generation may only manifest for prolonged periods^34^.

Generally, there were no differences in the benthic communities' richness, abundance, biomass, or even species taxonomical and functional composition when comparing the initial and final 24-day experimental period, nor when comparing the heating treatments. So, we could not conclude on a survival effect and species-specific or traits-specific differences due to the warming, as observed in empirical and longer-term studies^17,24,37,54^. Nevertheless, the Simpson index (measured with biomass) was lowest in the long heatwave treatment. We also found a trend for these samples to cluster due to the higher biomass of particular species and particular traits, i.e., *S. plana*.

These results may also reflect low replication, which has effectively detected patterns with artificial assembled communities before^33,34,55^. Nevertheless, for natural communities, a higher replication is desirable to account for natural variability and ascertain patterns^35^. As mentioned above, an opposite response was observed when comparing the weekly difference in bioturbation between the short and long term heatwave treatments. This result could be attributed to the slight differences in the community-specific and functional composition (e.g., the dominance of *S. plana* and associated traits) and behaviour during the heatwave and after its occurrence.

### Potential pitfalls and recommendations

Overall, in this pilot study, we found differences in bioturbation and nutrient dynamics attributed to the benthic communities' responses to warming. However, the inconsistent results regarding the effect of the tested heatwaves warrant further study and emphasise the complexity and unpredictability of warming effects in the natural biological communities. For this benthic fauna, from temperate southern regime subjected to wide daily temperature amplitudes, we did not find a clear directional shift in the response towards the warming exposure, emphasising the possible tolerance of the species in the study but also some of its vulnerabilities. The study was short-termed to infer a clear impact or conclude on the recovery period’s effectiveness from the heatwaves and potential communities’ resilience. Extending such experiments and testing more temperature scenarios seems a relevant forthcoming step. No doubt, heatwaves are increasing and may present different and unpredictable characteristics^1,19^. This study highlights the importance of studying temperature variability over average temperature changes^5^ and understanding its extremes' impact regarding duration and severity, seasonal timing, and frequency (incl. recurrence and diurnal variability^37^), in line with predictions^1,2,19^. It is also relevant to understand the factors that might influence the recovery of biological populations, such as temperature drop back to tolerable levels, its duration, and intermittency with the warming extremes and frequency, that could act as a climate refugia and might be critical to intertidal fauna^6^.

The present results also indicate that there might be community-specific responses to warming, which can be associated with species physiological responses^6,22^ and their functional roles in the context analysed^37,39,51^. Although the contribution of these individual species for bioturbation is relatively understood^27,38^, their behaviour within natural communities and under temperature variability may change due to the effect of the habitat itself, other abiotic constraints^51^ as tidal cycle and hydrodynamic stress^29,44^, species interactions^5^ or even due to an adaptive behaviour^37^. So, experimental community studies are needed to understand the implications of climate variability^37^. Nevertheless, testing all the above factors with natural communities with sufficient experimental replication is extremely challenging^56^. So efforts should privilege a set of conditions that can be scalable and used to increase our understanding of the role of benthic communities for mitigation or adaptation possibilities towards climate change impacts^27^. Experimental should include a focus on climate variability, with sufficient time scale and replication, and the actual contribution of these benthic organisms as part of a broader conservation strategy for maintaining healthy ecosystem functioning.

## Material and methods

### Sampling and experimental set-up

Biological samples were collected in the intertidal polyhaline flats of the Mondego estuary' south arm (40°9′47.91ʺN, 8°40′12.42ʺW) in October 2018. Muddy sediments characterise this area (∼80% sand; ∼20% silt) with ∼6.2% organic matter content^54^ and a water-flow velocity varying within 1.2 to 1.4 m s^−1^^24^. The average minimum and maximum air temperature ranged between 11.5 to 22.6 °C in October 2018 (www.ipma.pt). Usually, water temperature during this time varies around 17 °C^57^.

Twenty intact cores were collected with sediment and benthic communities, up to 14 cm depth, and gently placed in aquaria (12 cm × 12 cm × 40 cm). Excess bottom sediment was transformed into a slurry and positioned in the aquaria borders to fulfil its area up to 12 cm and ensure no air gaps, yet this represented less < 5% of the final sediment volume and was left to settle during the acclimation phase. UV- sterilised and pre-filtered (10 μm) seawater (salinity 34.7) was added to each aquarium up to 36 cm (volume of 2.88 L) and left for 48 h to settle down the sediment and for acclimation of the experimental procedures. After this time, the water was replaced once to remove the effect of nutrient pulses associated with assembly and ensure that changes in nutrients could be attributable to species activity^33^. The experimental period started 24 h later, and water no longer changed.

### Experimental design

We tested three temperature treatments, assuming five replicates per treatment, considering a (1) control, with a constant temperature of 17 °C, hereafter named as constant 17 °C; (2) short heatwave, with an 8 °C temperature increase for seven days, and a recovery period of 14 days when temperature decreased to 17 °C; (3) long heatwave, with 8 °C increase for 14 days and a recovery period of 7 days. The constant temperature of 17 °C was chosen based on the average values for the Mondego estuary^57^ and those verified at the sampling time^58^. Daily temperature amplitude may change with the effect of the tides, absence of light, and season. According to the Portuguese Institute for Sea and Atmosphere (IPMA), a heatwave occurs when the air temperature increases more than 5 °C compared to the climate baseline of the past 30-years for at least six consecutive days (www.ipma.pt)^4^. This temperature may translate into an increase in the intertidal water pools of more than 8 °C when exposed to temperature extremes (^40^ and unpublished data).

For each temperature treatment, the aquaria were placed in a water bath where the temperature was controlled with a control system (Aqua Medic AT Control-SW, version 9.0), according to the temperature treatments design. All aquaria were permanently aerated to ensure oxygen levels and kept under 12 h–12 h light/dark conditions. No food supplies were given to the aquaria. Oxygen, pH, salinity and temperature were measured to monitor any abnormal event during the experimental period, which lasted 24 days. Water samples were taken to quantify nutrient (nitrate and phosphate) release to the water column at days 0, 3, 10, 17 and 24. After 24 days, all aquaria were analysed to evaluate their macrobenthic communities. Additional five replicates were taken to assess the macrobenthic communities diversity, abundance, and biomass at the beginning of the experimental procedure, after the acclimation period (day 0), hereafter named as initial control.

### Sediment particle reworking

The extent of particle reworking was measured using fluorescent sediment profile imaging (f-SPI), following^59^. Luminophores that fluoresce under UV light (125–250 µm diameter, green colour; Brian Clegg, Ltd, UK) were added to the aquaria one day before the beginning of the experiment (day-1) when water was replaced, and every week (20 g), until the end of the experiment. We evaluated the distribution of luminophores per time with high-resolution images from one of the sides of the aquaria, where new luminophores were mostly placed every week. These were taken with a Canon EOS Rebel T5, 18 mm lens, set for an exposure of 10 s, f = 6.3 aperture and a film equivalent speed (light sensitivity) of ISO 200. The images were taken on the same days of the water samples collection, i.e., on days 0, 3, 10, 17 and 24. Then, images were analysed using a custom-made plugin ran within IMAGEJ (Version 1.48c, available at http://imagej.nih.gov/ij/, more details in^55,60^. Different descriptors of particle reworking were quantified to get a more accurate measurement of the bioturbation process^55^: (1) surface boundary roughness (SBR), representing the distance between the highest and lowest points of the sediment surface, used as a proxy on surficial activity; (2) mean luminophores' depth (Lmean), the mean depth of mixing of the luminophores; (3) median luminophores' depth (Lmedian), typical short-term depth of mixing depths; and (4) luminophores' maximum depth of mixing (Lmax), a punctual measure, reflecting the maximum extent of mixing over the long-term^55^. These particle reworking data were also analysed as the absolute weekly difference, i.e., the difference between the final and initial day for every seven days (day 10–day 3, day 17–day 10, and day 24–day 17), per temperature (17 °C, 25 °C and the drop from 25 °C to 17 °C) and treatments (constant temperature, short and long wave treatments.

Salinity, temperature, pH and oxygen levels in all aquaria were measured every three days to depict any abnormal event during the experimental procedure. For nutrients, all samples were filtered (nitrate cellulose filters, 0.45 μm porosity). Dissolved phosphate was analysed following the methods described in^61^. Nitrate was quantified by an adaptation of the spongy cadmium reduction technique^62^, subtracting nitrite value from the total.

### Benthic communities

All macrobenthic communities from initial and final experimental samples (5 replicates per treatment) were washed through 500 μm mesh sieves. Benthic organisms were sorted and identified up to species level with taxonomic guides^63^, counted, and their biomass quantified as ash-free dry mass (AFDM), by weighting dried (48 h at 60 °C) and combusted material (8 h at 450 °C).

The benthic communities were further analysed as a function of specific species traits, known to affect bioturbation^38^ and potentially nutrient release. These were body size measured as aquaria's average body size (numerical trait), and, as categorical traits, mobility (i.e., free movement, slow free movement, limited movement and fixed), sediment reworking type (i.e., epifauna, biodiffusors, conveyors, surficial modifiers and regenerators), feeding type (i.e., predator, deposit, grazer/deposit, interface and suspension feeders) and trophic level (i.e., carnivore, detritivore, herbivore and omnivore). Body size mediates structuring interactions among all traits. Mobility and sediment reworking types describe the species-sediment mixing^38^ that promote sediment oxygenation and nutrient fluxes^49^. Feeding type and trophic group describe the feeding behaviour and potential diet, affecting nutrient dynamics. The species classification as traits was obtained using data from online databases and literature (e.g., BIOTIC-http://www.marlin.ac.uk/biotic^38^, the complete classification of species is available in supplementary material).

### Data analyses

The particle reworking (SBR, Lmean, Lmedian, Lmax) and nutrient concentrations (phosphates and nitrates) data were tested with a PERMANOVA using a crossed design for the fixed factors temperature treatment (three levels: constant 17 °C, short heatwave, and long heatwave) with time as a repeated measure^64^. As the analyses were done for each parameter alone (particle reworking and nutrients), we treated each time as a separate variable. We then performed a multivariate analysis among treatments, using the Euclidean distance as resemblance matrix^64^.

The benthic communities of each aquarium were analysed as a function of species number, Simpson diversity, total abundance and total biomass. These univariate data were further explored with ANOVA for the factor temperature treatment. Then, the species' biomass data were square rooted and converted into a Bray–Curtis similarity matrix and analysed with PERMANOVA for the factor temperature treatment and with a PCO to visualise differences among treatments^64^. Finally, functional diversity was evaluated as Community Weighted Means (CWM) for each species' traits, as detailed above, per aquarium. CWM's express the dominant traits in a community, taking into account their relative biomass^65^, which for the categorical traits provide the relative percentage of each trait category in the communities. These were multiplied by the community biomass since this varied among aquaria. These trait data were also explored with PERMANOVA and PCO.

We checked the assumptions of the ANOVA and linear models with graphical procedures^66^. Analyses and graphs were done using R statistical and programming environment^67^, using the packages vegan^68^, FD^69^, ggplot2^70^, and software PRIMER v6 with PERMANOVA add-on routines^64^.

## Supplementary Information

Supplementary Information.
