# Supplementary material for: Benthic estuarine communities' contribution to bioturbation under the experimental effect of marine heatwaves
Source: Sci Rep. 2021 Jun 1;11:11422. doi: 10.1038/s41598-021-90720-7 (PMC8169769; doi:10.1038/s41598-021-90720-7)
Supplement: Supplementary file 1 — Supplementary Information. [file 41598_2021_90720_MOESM1_ESM.docx]

**Supporting information**

**Benthic estuarine communities’ contribution to bioturbation under the experimental effect of marine heatwaves**

Short title: Estuarine benthic bioturbation under warming extremes

Dolbeth M^1*^, Babe O^1^, Costa DA^1,2^, Mucha AP^1^, Cardoso PG^1^, Arenas F^1^

^1^ CIIMAR - Interdisciplinary Centre of Marine and Environmental Research, Novo Edifício do Terminal de Cruzeiros do Porto de Leixões, Avenida General Norton de Matos s/n, 4450-208 Matosinhos, Portugal

^2^ UFPB - Federal University of Paraíba, Center of Exact and Nature Sciences (CCEN), Department of Systematics and Ecology (DSE), Jardim Cidade Universitária s/n, 58051-090 João Pessoa, Brazil

* corresponding author: mdolbeth@ciimar.up.pt

**Table 1S.** Species found in the aquaria, with the indication of the measured species average body mass trait (in ash-free dry mass - AFDM) within the aquaria, and remaining trait categories defined according to the literature.

| Species | Body mass mg AFDM | Mobility | Sediment Reworking | Feeding | Trophic group |
| --- | --- | --- | --- | --- | --- |
| *Amphisbetia distans* | 0.7 | Fixed | Epifauna | Predator/Scavenger | Carnivore |
| *Capitella capitata* | 1.9 | Limited movement | Conveyors | Deposit | Detritivore |
| *Carcinus maenas* | 247.6 | Free movement | Regenerator | Predator/Scavenger | Omnivore |
| *Cerastoderma edule* | 15.1 | Limited movement | Surficial modifiers | Suspension | Detritivore |
| *Corophium volutator* | 1 | Free movement | Surficial modifiers | Interface | Detritivore |
| *Cyathura carinata* | 0.8 | Limited movement | Surficial modifiers | Predator/Scavenger | Omnivore |
| *Hediste diversicolor* | 132.8 | Free movement | Biodiffusors | Predator/Scavenger | Omnivore |
| *Nephtys cirrosa* | 0.7 | Slow free movement | Biodiffusors | Predator/Scavenger | Carnivore |
| *Peringia ulvae* | 6.7 | Slow free movement | Surficial modifiers | Grazer/Deposit | Detritivore |
| *Scrobicularia plana* | 1422.6 | Limited movement | Surficial modifiers | Interface | Detritivore |
| *Sphaerosyllis hystrix* | 0.3 | Slow free movement | Surficial modifiers | Predator/Scavenger | Carnivore |
| *Tanais dulongii* | 0.7 | Limited movement | Surficial modifiers | Grazer/Deposit | Omnivore |

**Table 2S.** Summary of significant terms from the PERMANOVA analyses, using sediment particle reworking variables and nutrient concentrations as dependent variables, and temperature treatments (Control, Short heatwave, Long heatwave) and time (day 0, 3, 10, 17 and 24) as explanatory variables, with the indication of the all pairwise comparisons (non-significant ones in grey). Tested terms were: single ones - Temperature Time; 2-way interaction – Temperature x Time. HW, heatwaves; U. perm, unique permutations

| Dependent variable | Main test | | | | | | Pairwise tests | | |
| --- | --- | --- | --- | --- | --- | --- | --- | --- | --- |
|  | **Significant terms** | **d.f.** | **Pseudo-F** | **P-perm** | **U. perm** | **Terms/levels of factor** | | **P-perm** | **U. perm** |
| SBR | none | 4 | n.s | >0.267 | 999 | - | | - | - |
| Lmean | Time | 4 | 12.52 | 0.001 | 998 | Day 0, Day 3  Day 0, Day 10  Day 0, Day 17  Day 0, Day 24  Day 3, Day 10  Day 3, Day 17  Day 3, Day 24  Day 10, Day 17  Day 10, Day 24  Day 17, Day 24 | | 0.039  0.001  0.001  0.001  0.019  0.001  0.001  0.322  0.057  0.325 | 997  998  996  997  999  996  995  999  998  998 |
| Lmedian | Time | 4 | 12.194 | 0.001 | 999 | Day 0, Day 3  Day 0, Day 10  Day 0, Day 17  Day 0, Day 24  Day 3, Day 10  Day 3, Day 17  Day 3, Day 24  Day 10, Day 17  Day 10, Day 24  Day 17, Day 24 | | 0.398  0.002  0.001  0.001  0.002  0.001  0.001  0.211  0.019  0.16 | 997  998  995  998  999  996  995  998  997  998 |
| LMax | Time | 4 | 8.1727 | 0.001 | 995 | Day 0, Day 3  Day 0, Day 10  Day 0, Day 17  Day 0, Day 24  Day 3, Day 10  Day 3, Day 17  Day 3, Day 24  Day 10, Day 17  Day 10, Day 24  Day 17, Day 24 | | 0.007  0.004  0.001  0.001  0.498  0.119  0.049  0.361  0.138  0.633 | 997  996  993  995  997  996  994  996  995  994 |
| ABS Dif SBR | none | 1-2 | n.s | >0.26 | 999 |  | |  |  |
| ABS Dif Lmean | Temperature*Time | 1 | 7.6063 | 0.010 | 999 |  | |  |  |
| ABS Dif Lmedian | Temperature*Time | 1 | 6.7660 | 0.015 | 999 |  | |  |  |
| ABS Dif Lmax | none | 1-2 | n.s | >0.096 | 999 |  | |  |  |
| PO_4_-P | Temperature | 2 | 3.4786 | 0.054 | 999 | Control, Short HW  Control, Long HW  Short HW, Long HW | | 0.034  0.026  0.1741 | 998  997  999 |
|  | Time | 4 | 3.9626 | 0.017 | 999 | Day 0, Day 3  Day 0, Day 10  Day 0, Day 17  Day 0, Day 24  Day 3, Day 10  Day 3, Day 17  Day 3, Day 24  Day 10, Day 17  Day 10, Day 24  Day 17, Day 24 | |  |  |
| NO_3_-N | Temperature x Time | 8 | 2.4883 | 0.045 | 999 | None considered significant, yet having a low number of possible permutations for the interaction (due to low nr of replicates) | | >0.098 | 7 to 10 |

**Table 3S.** Summary of the ANOVA (univariate data and parametric data, F-value and p-value) and PERMANOVA (multivariate data, Pseudo-F and P-perm) analyses, using benthic communities as dependent variables and temperature treatments (Control, Short heatwave – HV, Long HW) as explanatory variables, with the indication of the significant results with an * and pairwise tests, when significant.

| Dependent variable | d.f. | F-value/  Pseudo-F | p-value/  P-perm | Pairwise tests | p-value |
| --- | --- | --- | --- | --- | --- |
| Number of species | 3 | 1.1754 | 0.3501 | n.s. | n.s. |
| Simpson index (biomass) | 3 | 4.1096 | 0.02436* | Control, Constant 17ºC  Control, ShortHW  Control, LongHW  Constant 17ºC, ShortHW  Constant 17ºC, LongHW  ShortHW, LongHW | 0.9993171  0.2048226  0.5209947  0.1676061  0.5931809  0.0152274* |
| Community density | 3 | 0.3297 | 0.804 | n.s. |  |
| Community Biomass | 3 | 1.8135 | 0.1853 | n.s. |  |
| Benthic communities composition | 3 | 0.63337 | 0.72 | n.s. |  |
| Traits communities composition | 3 | 0.83137 | 0.532 | n.s. |  |

**Table 4S.** Dominant body size and other traits categories within the community for each temperature treatment (sample), according to the community weighted mean analyses. These values represent the community's dominant traits, taking into all individuals. For the quantitative trait body mass, the value refers to the average individual body mass measured in the community per sample (aquarium). For the remaining categorical traits, the dominant traits are measured as a percentage (e.g. in the initial control 1^st^ sample, the average body mass of the individuals that compose the community was 35.1 mg AFDW, 82 % had slow, free movement, 93.7% were surficial modifiers, 82% were grazers/deposit and 94 detritivores.

| *Treatment* | *Body Mass (mg AFDM)* | *Mobility* | *%* | *Sediment Reworking* | *%* | *Feeding* | *%* | *Trophic group* | *%* |
| --- | --- | --- | --- | --- | --- | --- | --- | --- | --- |
| Initial control | 35.1 | Slow Free Movement | 82.0% | Surficial Modifiers | 93.7% | Grazer/Deposit | 82.0% | Detritivore | 94.0% |
|  | 1374.4 | Limited Movement | 96.6% | Surficial Modifiers | 99.7% | Interface | 96.6% | Detritivore | 99.7% |
|  | 434.5 | Slow Free Movement | 68.0% | Surficial Modifiers | 98.2% | Grazer/Deposit | 68.0% | Detritivore | 98.2% |
|  | 32.1 | Slow Free Movement | 91.1% | Surficial Modifiers | 95.5% | Grazer/Deposit | 91.1% | Detritivore | 96.4% |
|  | 1098.9 | Limited Movement | 77.2% | Surficial Modifiers | 95.3% | Interface | 76.7% | Detritivore | 95.3% |
| Constant 17ºC | 1342.8 | Limited Movement | 96.3% | Surficial Modifiers | 100.0% | Interface | 94.4% | Detritivore | 100.0% |
|  | 8.4 | Slow Free Movement | 98.6% | Surficial Modifiers | 98.7% | Grazer/Deposit | 98.6% | Detritivore | 98.7% |
|  | 1140.5 | Limited Movement | 80.2% | Surficial Modifiers | 100.0% | Interface | 80.1% | Detritivore | 99.9% |
|  | 837.9 | Limited Movement | 58.2% | Surficial Modifiers | 91.6% | Interface | 58.0% | Detritivore | 91.5% |
|  | 1144.0 | Limited Movement | 80.4% | Surficial Modifiers | 99.7% | Interface | 80.3% | Detritivore | 99.7% |
| Short Heatwave | 456.1 | Slow Free Movement | 64.0% | Surficial Modifiers | 96.2% | Grazer/Deposit | 64.0% | Detritivore | 96.2% |
|  | 1152.3 | Limited Movement | 82.0% | Surficial Modifiers | 99.8% | Interface | 80.9% | Detritivore | 99.8% |
|  | 912.9 | Limited Movement | 65.7% | Surficial Modifiers | 100.0% | Interface | 64.0% | Detritivore | 100.0% |
|  | 207.2 | Slow Free Movement | 65.7% | Surficial Modifiers | 97.3% | Grazer/Deposit | 65.7% | Detritivore | 97.4% |
|  | 949.1 | Limited Movement | 71.2% | Surficial Modifiers | 99.4% | Interface | 66.5% | Detritivore | 99.4% |
| Long Heatwave | 1297.4 | Limited Movement | 91.9% | Surficial Modifiers | 99.8% | Interface | 91.1% | Detritivore | 99.8% |
|  | 1317.6 | Limited Movement | 92.5% | Surficial Modifiers | 99.3% | Interface | 92.5% | Detritivore | 99.3% |
|  | 29.2 | Slow Free Movement | 96.1% | Surficial Modifiers | 99.9% | Grazer/Deposit | 96.1% | Detritivore | 99.9% |
|  | 1228.4 | Limited Movement | 86.3% | Surficial Modifiers | 99.8% | Interface | 86.3% | Detritivore | 99.8% |
|  | 1308.0 | Limited Movement | 91.9% | Surficial Modifiers | 99.8% | Interface | 91.9% | Detritivore | 99.8% |
